# Morphological Influences and Energetic Walking Flexibility in Determining Preferred vs. Optimal Speeds: An Evolutionary Human Ecology Perspective on Children and Adolescents

**DOI:** 10.1002/ajpa.70152

**Published:** 2025-11-07

**Authors:** Guillermo Zorrilla‐Revilla, Olalla Prado‐Nóvoa, Kevin P. Davy, Rebeca García‐González, Eleni Laskaridou, Kristen R. Howard, Elaina L. Marinik, José Miguel Carretero, Stella L. Volpe

**Affiliations:** ^1^ Laboratorio de Evolución Humana Universidad de Burgos Burgos Spain; ^2^ CIAS–Research Centre for Anthropology and Health University of Coimbra Coimbra Portugal; ^3^ Department of Education and Teacher Training, Faculty of Law, Education and Humanities Universidad Europea de Madrid Madrid Spain; ^4^ Department of Human Nutrition, Foods, and Exercise Virginia Polytechnic Institute and State University (Virginia Tech) Blacksburg Virginia USA; ^5^ Department of Human Nutrition, Foods, and Exercise, Human Integrative Physiology Laboratory Virginia Polytechnic Institute and State University (Virginia Tech) Blacksburg Virginia USA; ^6^ Graduate Program in Translational Biology, Medicine, and Health Virginia Polytechnic Institute and State University (Virginia Tech) Blacksburg Virginia USA

**Keywords:** anthropometry, children, human behavioral ecology, human bioenergetics, Hunter‐gatherers, optimal locomotion speed

## Abstract

**Objectives:**

Locomotion is fundamental to the survival of our species. The most comfortable walking speed may be the most efficient for allocating conserved energy for other functions. However, whether preferred (PLS) and optimal (OLS) speeds align in children and adolescents remains unclear. This study aimed to determine whether OLS and PLS are similar in children and adolescents and how anthropometry influences both speeds and their differences.

**Materials and Methods:**

Eleven females and 17 males (8–17 years of age) were anthropometrically characterized. Five treadmill walking pace tests were used to identify the OLS and U‐shaped relationship between energy expenditure and speed (*χ* ^2^ CoT), indicating walking flexibility. Additionally, PLS was self‐selected using the same protocol. Differences between OLS and PLS were calculated (mean difference [MD]).

**Results:**

No significant sex differences in anthropometry and speed‐related variables were found. OLS, PLS, and their MD in the pooled sample were 3.05 ± 0.13, 2.46 ± 0.51, and 0.60 ± 0.46, respectively, with significant differences between OLS and PLS (*p* < 0.0001). Femur length (FL), Bi‐iliac breadth (BIL), and *χ* ^2^ CoT explained variance in OLS, PLS, and MD, respectively, in the forward stepwise regression models.

**Discussion:**

Unlike adults, OLS and PLS are not interchangeable in children and adolescents. Participants with lower *χ* ^2^ CoT (greater flexibility) can select comfortable speeds farther from OLS without energetic penalty. Taller individuals with longer femurs and wider hips might have biomechanical advantages in reaching higher OLS and PLS, but this reduces flexibility. These traits, along with the growth and development pattern of *Homo sapiens* , may reflect evolutionary advantages relevant to interspecies competition.

**Summary**

- OLS and PLS are not interchangeable in children and adolescents.
- Morphology explains differences between optimal and preferred walking speeds.
- Walking flexibility may offer adaptive advantages in human evolution.

## 1 Introduction

Bipedal locomotion is central to human evolution (Lovejoy 1981, 1988; Malina and Little 2008; Sockol et al. 2007), having shaped our anatomy, physiology, and behavior over millions of years. Walking accounts for most human activity‐related energy expenditure (EE) (Bouterse 2017; Froehle, Yokley, and Churchill 2013; Passmore and Durnin 1955) and likely even more so in past subsistence activities (Barazesh and Ahmad Sharbafi 2020; King et al. 2015; Lakka and Laaksonen 2007; Majed et al. 2024; Malina and Little 2008; Owen et al. 2004; Pontzer 2012; Siegel et al. 1995; Steudel‐Numbers and Wall‐Scheffler 2009; Tudor‐Locke et al. 2009; Vidal‐Cordasco et al. 2021). As such, reducing time, EE, or distance walking may be critical for energy conservation (Kramer 2004).

Humans may choose a locomotion speed that minimizes EE per distance traveled when walking alone (Alexander 2002; Bouterse 2017; Browning and Kram 2005; Ralston 1958; Sparrow and Newell 1998; Vaughan 2003). This optimal locomotion speed (OLS) is the nadir of the U‐shaped relationship between walking speed and the metabolic cost of transport (COT). Importantly, some previous researchers have suggested that a wider U‐shaped relation (*χ* ^2^ CoT) increases the likelihood that the self‐selected or preferred locomotion speed (PLS) coincides with OLS (Anderson and Pandy 2001; Frohele, Yokley, and Churchill 2013; Ralston 1958; Wagnild and Wall‐Scheffler 2013). The OLS includes time, distance, and energy cost and, therefore, has become a useful parameter for modeling human behavior under the Human Behavioral Ecology framework (Bird and Bliege Bird 2000; Mateos et al. 2022; Wagnild and Wall‐Scheffler 2013; Wall‐Scheffler 2012b).

Although it is assumed that, throughout our evolutionary history and under selective pressure to conserve energy, individuals tend to choose or prefer a walking speed that is their most efficient walking speed (i.e., similar to OLS) (Selinger et al. 2015, but see Majed et al. 2024), few researchers have directly addressed this possibility (Gast et al. 2019; Gidley and Lankford 2021; Wall‐Scheffler 2015; Wall‐Scheffler and Myers 2013). Most of the existing research on this topic has focused on clinical populations (Abe et al. 2021; Browning et al. 2006; Browning and Kram 2005; Fernández Menéndez et al. 2019), with even fewer investigations involving non‐adult populations (under 18 years old) (Peyrot et al. 2012). Some (Browning and Kram 2005; Das Gupta et al. 2019; Ralston 1958; Zarrugh et al. 1974), but not all, previous studies (Gast et al. 2019; Hunter et al. 2010; Majed et al. 2024; Mohler et al. 2007; Willis et al. 2005) have indicated that healthy and adult populations prefer a walking speed energetically similar to their OLS. Whether PLS is similar to OLS in non‐adults with a healthy body weight is unknown.

OLS has been reported to be an important determinant of the sex and age composition of a human group engaged in foraging and mobility activities (Bouterse and Wall‐Scheffler 2018; Costa 2010; Wagnild and Wall‐Scheffler 2013; Wall‐Scheffler 2012a, 2012b). Similarly, walking with a partner of a different sex can alter the locomotion pace if one or both individuals' walking speeds deviate from their OLS (Bouterse and Wall‐Scheffler 2018; Wagnild and Wall‐Scheffler 2013). Interestingly, Mateos et al. (2022) and Zorrilla‐Revilla (2022) suggested that OLS of children and adolescents may not affect human groups' mobility and composition as previous researchers have hypothesized. The results of previous studies suggest that anthropometric characteristics such as body mass, limb length (greater tibia and femur length), and bi‐iliac breadth may impact OLS in both adults (Wall‐Scheffler 2012a, 2012b) and younger individuals (Zorrilla‐Revilla et al. 2024). However, the morphological factors determining human OLS have not been fully characterized.

The primary purpose of our study was to determine whether OLS and PLS differ from each other in a sample of children and adolescents, an understudied population. In addition, we aimed to assess the contribution of morphological factors to the variability in both optimal and preferred speeds, and to examine the possible mismatch between them. We discussed the potential anthropometric factors that influence different mobility variables and their ecological consequences in *Homo* species. We consider our findings in the context of the Human Life History evolution perspective within the Human Behavioral Ecology framework.

## 2 Material and Methods

### 2.1 Participants

Volunteers were recruited through advertisements in local media and social networks in Blacksburg, Virginia, USA, and nearby areas as part of a larger study. Twenty‐eight volunteers (11 females and 17 males, from 8 to 17 years of age) were involved. All the data were collected from May 2023 to December 2023. Participants and their respective legal guardians were informed about the nature of the study, and both verbal and written consent were obtained (assent was obtained from children and adolescents). All volunteers involved were healthy weight and normotensive individuals, who were free from metabolic, cardiorespiratory, renal, or neoplastic diseases. None of the participants were taking any medications that may have affected the variables studied. In addition, none of the participants had any physical disabilities, recent injuries, or other conditions that could have altered their physical activity. Our study was approved by the Virginia Tech Institutional Review Board (IRB #23–324).

### 2.2 Experimental Protocols

All volunteers were fasted overnight and avoided vigorous exercise for the previous 12 h. All participants were anthropometrically characterized based on the normalized standards of Lapunzina and Aiello (2002). Body mass (BM) and height were determined to the nearest 0.1 kg and 0.1 cm using an integrated stand‐on scale with stadiometer (Scale‐Tronix 5002, Skaneateles Falls, NY), respectively. Femur and tibial length (FL and TL, respectively) and bi‐iliac and bi‐trochanteric width (BIL and BiTR, respectively) were obtained as described in Zorrilla‐Revilla et al. (2024) (Table 1).

**TABLE 1:** Age, anthropometric, and OLS parameters in girls (♀) and boys (♂).

Variable	Pooled sample (*n* = 28)	♀ (*n* = 11)	♂ (*n* = 17)	*t*	*F*	*W*
Age (years)	12.79 (2.70)	12.91 (2.30)	12.71 (3.00)	0.19	0.59	84.50
Height (cm)	157.35 (18.30)	157.28 (9.09)	157.46 (22.66)	0.03	0.16**	82
BM (kg)	46.84 (13.33)	45.56 (8.33)	47.71 (16.12)	−0.41	0.27*	95
BIL (cm)	24.84 (2.55)	25.26 (1.65)	24.57 (3.02)	0.69	0.30	68
BiTR (cm)	28.26 (3.36)	28.45 (2.11)	28.13 (4.03)	0.24	0.27*	83
FL (cm)	38.08 (5.68)	38.58 (3.60)	37.76 (6.78)	0.37	0.28*	70.50
TL (cm)	36.49 (5.62)	36.41 (2.02)	36.55 (7.13)	−0.06	0.08***	82
*χ* ^2^ CoT	11.57 (3.29)	10.47 (0.95)	12.73 (3.95)	−1.85	0.06****	115
OLS (mph)	3.05 (0.13)	3.08 (0.11)	3.03 (0.14)	0.96	0.62	70.50
PLS (mph)	2.46 (0.51)	2.41 (0.48)	2.50 (0.54)	−0.45	0.77	93
MD (mph)	0.60 (0.46)****	0.67 (0.42)	0.54 (0.50)	0.71	0.68	62.50

*Note:* Data are expressed as mean ± standard deviation (SD). *χ* ^2^ CoT represents the quadratic coefficient. OLS and PLS refer to the optimal and preferred locomotion speeds, respectively. MD is the difference between OLS and PLS. Significance levels are indicated as follows: *p < 0.05, **p < 0.01; ***p < 0.001; ****p < 0.0001 for *t* tests (*t* comparing means), *F* tests (*F* comparing SD), and Mann–Whitney tests (*W*‐comparing medians). MD**** indicates significant differences in the means of OLS and PLS in the pooled sample (*n* = 28) (*t* = 6.23).

Abbreviations: BIL = bi‐iliac breadth; BiTR = bi‐trochanteric breadth; FL = femur length; TL = tibial length.

Walking trials were performed on a treadmill (COSMED T170, Chicago, IL) and EE was measured using indirect calorimetry (Parvo Medics, TrueOne 2400 Metabolic Measurement System; OUSW 4.3.4; Murray, Utah, USA). EE (kilocalories) was calculated using the Weir equation (Weir 1949).

Each participant completed a single locomotion test on the treadmill after a brief familiarization period. Walking was performed for 5 min at each of the five different speeds, with 1 min resting between stages. Randomization of the order of speeds (1.24, 1.8, 2.4, 3.1, and 3.7 miles per hour [mph]) was performed using a random number generator (Random.org). The selected range of speeds contained the optimal speed range proposed for adults (2.46–3.09 mph) (Gomeñuka et al. 2014; Steudel‐Numbers and Tilkens 2004; Wagnild and Wall‐Scheffler 2013; Wall‐Scheffler 2012b; Waters et al. 1988) as well as one faster speed and two slower speeds. These ranges were similar to those previously used in children and adolescents (DeJaeger et al. 2001; Smeby 2017; Zorrilla‐Revilla 2022).

During a second laboratory session, each volunteer walked at a comfortable speed (PLS). PLS was the speed freely selected by each participant following instructions to walk at a comfortable pace, mimicking the speed they might select while walking outdoors. OLS was the speed determined from the initial laboratory session (Zorrilla‐Revilla et al. 2024). OLS was developed as the second test to avoid influencing the selection of the preferred speed. Each walking test lasted 5 min, followed by a 1‐min rest period. Participants were instructed not to hold onto the treadmill handrail to avoid altering the pattern and energy cost of walking (Meyns et al. 2013; Stephenson et al. 2009; Zahradka 2010). The last 3 min of each stage were used for calculations, as suggested by several researchers (Browning and Kram 2005; Mateos et al. 2022; Usherwood and Bertram 2003; Yamasaki et al. 1991). This ensured that a steady state in oxygen consumption was achieved. Two participants did not complete the second session and were removed from analyses that relied on PLS.

*StatGraphics Centurion XVI.I* (The Plains, Virginia) and *Excel 2019* software (Microsoft Corporation) were used for statistics, and the significance was set a priori at *p* < 0.05. Descriptive statistics were conducted for the anthropometric, PLS, and OLS‐related variables (*χ* ^2^ CoT), dichotomizing the sample by sex. Results were expressed as mean and standard deviation (SD). Gross CoT expresses energy per unit distance. Gross CoT was calculated as gross metabolic power (kcal/h) divided by treadmill speed V (mph), yielding CoT in kcal/mile. For each participant, we fit a second‐order polynomial to the CoT–speed data across the five randomized paces following prior work (Mateos et al. 2022; Steudel‐Numbers and Wall‐Scheffler 2009; Zorrilla‐Revilla 2022):

(1) $$\mathrm{CoT}\left(V\right)=a\cdot V^{2}-\mathrm{bV}+c$$

The OLS corresponds to the vertex of this parabola and is obtained by setting the first derivative to zero:

(2) $$d\mathrm{CoT}/\mathrm{dV}=2\mathrm{aV}-b=0\Rightarrow \mathrm{OLS}\;\left(\mathrm{mph}\right)=b/\left(\text{2a}\right)$$

The minimum CoT (minCoT) is CoT evaluated at the OLS. *χ* ^2^ CoT was defined as the quadratic coefficient (*a*) in Equation (1), which characterizes the curvature (shape) of the U‐shaped CoT–speed relation: larger *a* values indicate a narrower parabola and, therefore, a greater energetic penalty for departures from OLS (i.e., lower walking flexibility). Because it serves as a shape descriptor, *χ* ^2^ CoT does not encode absolute EE; those magnitudes are provided by CoT (kcal/mile).

The mean difference (MD) was calculated as the difference between OLS and PLS. Normality of data distributions was assessed using the Kolmogorov–Smirnov test (*p* < 0.05). Where parametric assumptions were met, independent *t* tests were used to compare group means and *F* tests were used to compare their standard deviations. When appropriate, the Mann–Whitney *U* test was applied to compare medians as a conservative validation of *t* test findings. Forward stepwise regressions were used to determine whether independent variables such as age, body height, BM, FL, TL, BIL, BiTR, and sex influenced the PLS, the OLS, and the MD. These independent variables, some of which are relevant for explaining key aspects of locomotor biomechanics, have been used by previous researchers with similar objectives (Mateos et al. 2022; Vidal‐Cordasco et al. 2017; Wall‐Scheffler 2012b; Zorrilla‐Revilla et al. 2020).

## 3 Results

All the variables were normally distributed. Therefore, parametric tests were used for group comparisons. Additionally, the Mann–Whitney *U* test supported the findings of the *t* tests, revealing no statistically significant differences between sexes in any of the variables regarding means (*t*) or medians (*W*) (Table 1); however, girls were, on average, older (12.91 vs. 12.71 years of age), shorter (157.28 vs. 157.46 cm) and lighter (45.56 vs. 47.71 kg), with larger FL values (38.58 vs. 37.76 cm) and shorter TL values (36.41 vs. 36.55 cm), greater BIL values (25.26 vs. 24.57 cm), greater BiTR values (28.45 vs. 28.13 cm), higher OLS (3.08 vs. 3.03 mph) and lower *χ* ^2^ CoT (10.47 vs. 12.73 *a*) compared to boys. The SDs of age, BIL, OLS, PLS, and MD were not significantly different between them. In contrast, the SDs for boys' height, BM, BiTR, FL, TL, and *χ* ^2^ CoT were significantly higher than those for girls (Table 1).

Due to the lack of significant differences between the means and medians, the sample was treated homogeneously for the subsequent statistical analyses. For the overall sample, a significant difference was observed between OLS and PLS (*p* < 0.0001), with a mean difference of 0.6 mph between them. Notably, OLS exhibits higher values than PLS in the pooled sample.

**FIGURE 1:**
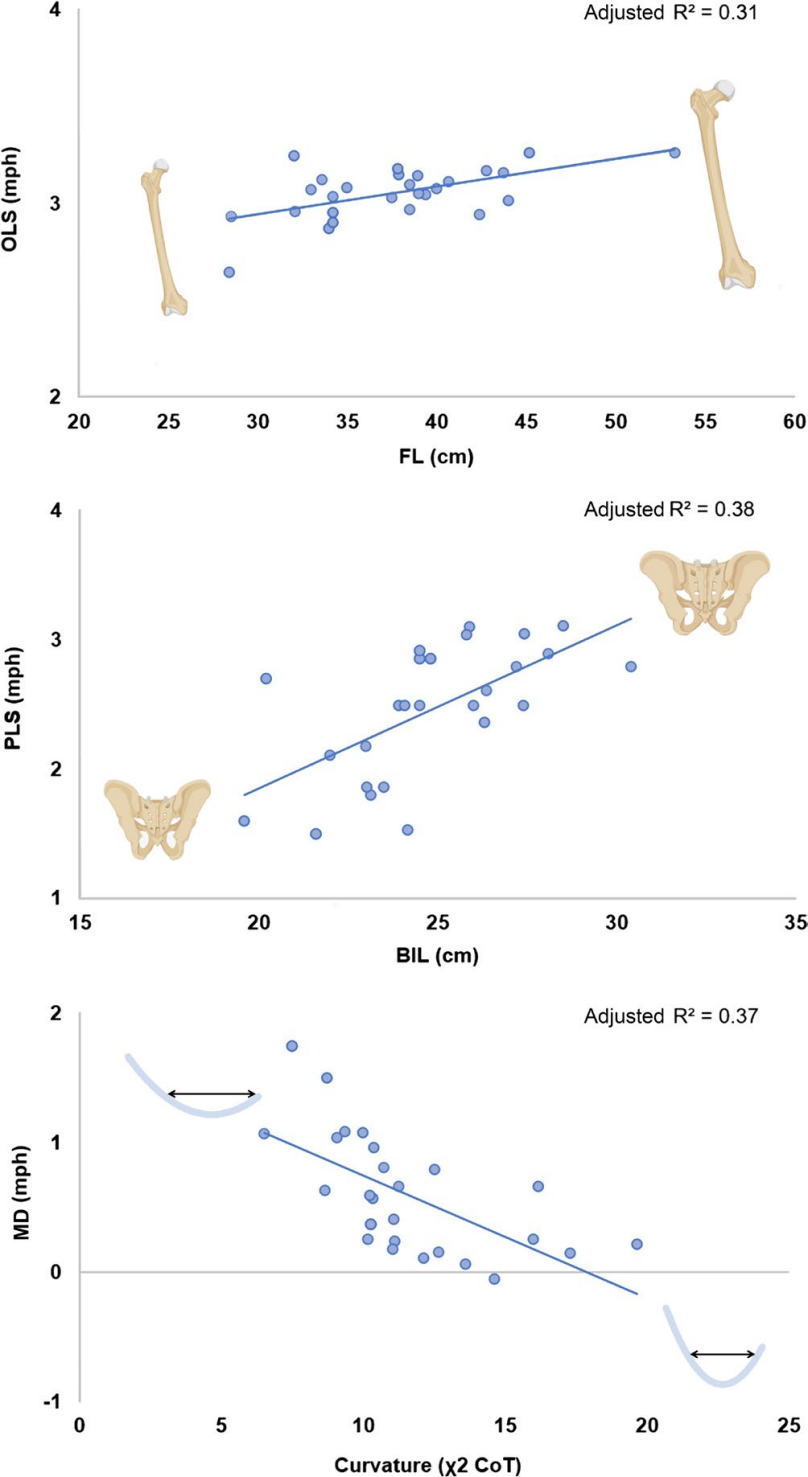
Significant associations between FL, BIL, *χ* ^2^ CoT, and OLS, PLS, and MD, respectively. The graphics display the only variable approaching statistical significance via forward stepwise multiple regression. OLS and PLS refer to optimal and preferred locomotion speeds, respectively. MD is the difference between OLS and PLS (MD = OLS − PLS), expressed in miles per hour (mph). *R* ^2^ refers to adjusted *R* ^2^. BIL (bi‐iliac breadth) and FL (femur length) are expressed in centimeters (cm). *χ* ^2^ CoT represents the quadratic coefficient of the cost of transport (COT) curve, which reflects the curvature of the parabolic relationship between speed and energy cost.

On the other hand, FL showed a positive correlation with OLS and was the only variable that remained in the model, accounting for 31% of its variability (adj. *R* ^2^; see Table 2). BIL accounted for 38% of the variability in PLS (adj. *R* ^2^) with no other significant predictors. Likewise, only *χ* ^2^ CoT was negatively correlated with MD, accounting for 37% of the variability (adj. *R* ^2^) (Figure 1).

**TABLE 2:** Predictive stepwise multiple linear regressions.

Predicted variable		Predictor variables	*p*	Linear regression models	adj. *R* ^2^	*p*
MD	*a*	*χ* ^2^ CoT	< 0.01	1.69973–0.0951597 × *χ* ^2^ CoT	0.37	< 0.01
		Age (years)	> 0.05			
		Sex (0 = girl; 1 = boy)	> 0.05			
		FL (cm)	> 0.05			
		Body height (cm)	> 0.05			
		BM (kg)	> 0.05			
		TL (cm)	> 0.05			
		BIL (cm)	> 0.05			
		BiTR (cm)	> 0.05			
OLS	*b*	FL (cm)	< 0.01	2.5123 + 0.0143608 × FL	0.31	< 0.01
		Age (years)	> 0.05			
		Sex (0 = girl; 1 = boy)	> 0.05			
		Body height (cm)	> 0.05			
		BM (kg)	> 0.05			
		FL (cm)	> 0.05			
		TL (cm)	> 0.05			
		BiTR (cm)	> 0.05			
		*χ* ^2^ CoT	> 0.05			
PLS	*c*	BIL (cm)	< 0.001	−0.691006 + 0.126868 x BIL	0.38	< 0.001
		Age (years)	> 0.05			
		Sex (0 = girl; 1 = boy)	> 0.05			
		Body height (cm)	> 0.05			
		BM (kg)	> 0.05			
		FL (cm)	> 0.05			
		TL (cm)	> 0.05			
		BiTR (cm)	> 0.05			
		*χ* ^2^ CoT	> 0.05			

*Note:* Three different linear regression models are presented for MD, OLS, and PLS. Only variables shaded in gray were selected by the models. *χ* ^2^ CoT represents the quadratic coefficient. OLS and PLS refer to optimal and preferred locomotion speeds, respectively. MD is the difference between OLS minus PLS. *R* ^2^ refers to the adjusted model. Age is expressed in years. Sex is expressed as: Sex = 0 and sex = 1, representing girls and boys, respectively.

Abbreviations: BM = body mass; BIL = bi‐iliac breadth; BiTR = bi‐trochanteric breadth; FL = femur length; TL = tibial length—all expressed in centimeters (cm).

## 4 Discussion

### 4.1 Findings on PLS and OLS in Children and Adolescents

**FIGURE 2:**
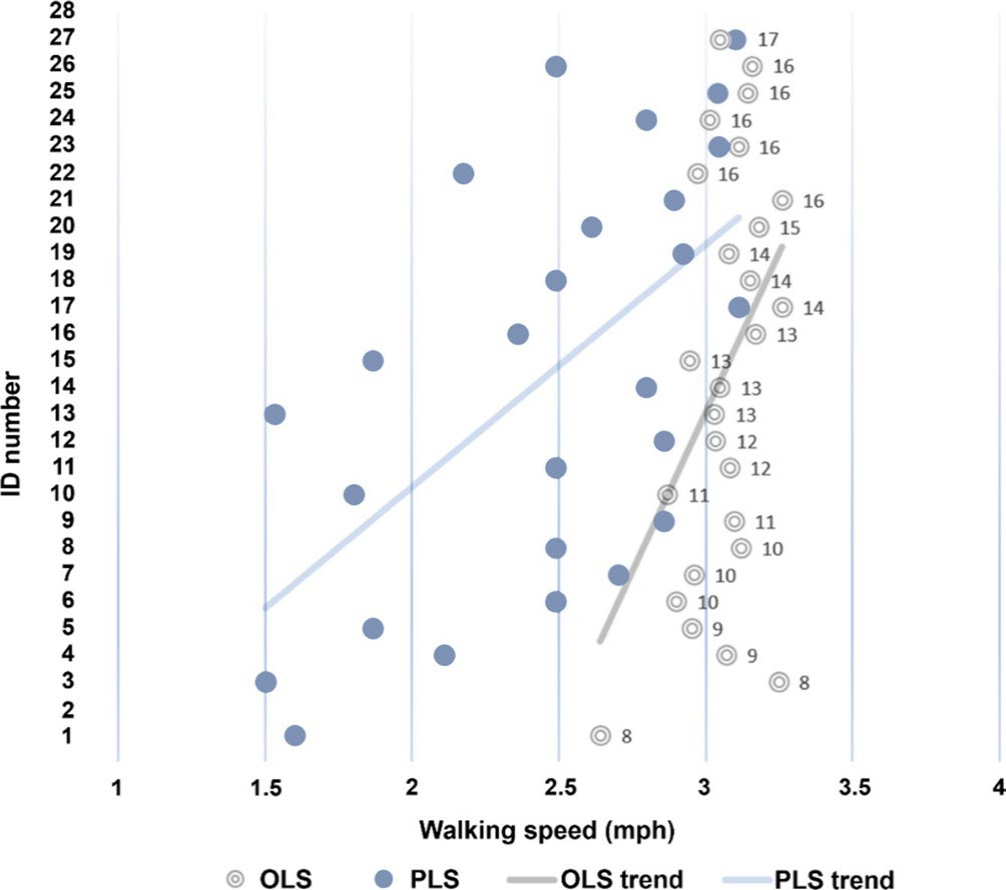
Distances (MD) between PLS and OLS. OLS and PLS refer to optimal and preferred locomotion speeds, respectively. PLS (blue) and OLS (gray) plots illustrate visual disparities between the preferred and optimal locomotion speeds for each participant (identified by ID number). The blue and black lines describe the positive trends observed in PLS and OLS, respectively. The dots are arranged in ascending order of age (numbers next to dots), from bottom to top. Mph refers to miles per hour.

The key finding of our study is that children and adolescents did not self‐select their OLS in the same manner as their PLS. Therefore, PLS and OLS cannot be used interchangeably in our sample. Our results are consistent with those of Majed et al. (2024), but diverge from the findings of Fernández Menéndez et al. (2019) and Gast et al. (2019), although the latter researchers conducted their studies in adult populations. Interestingly, Figure 2 shows that as participants' age increases, the differences between PLS and OLS decrease. Nevertheless, in our stepwise multiple regression analyses, MD showed a positive correlation with the amplitude of the parabola, discarding the rest of the independent variables (age, sex, height, TL, FL, BIL, and BiTR). This means that higher *χ* ^2^ CoT values (indicating less walking flexibility) lead to smaller differences between PLS and OLS and vice versa. Our present outcomes are reinforced and complemented by the findings of Zorrilla‐Revilla et al. (2024), who found that higher stature and longer body segments in children and adolescents were positively correlated with greater parabola narrowness (higher *χ* ^2^ CoT values). This suggests that taller individuals exhibit lower efficiency when walking away from their optimal walking speed (Zorrilla‐Revilla et al. 2024). Consequently, individuals with a broader amplitude of the parabola, typically the shortest children, can afford to choose PLSs that are further away from their OLS (high MD) without experiencing significant energy penalties. Conversely, those with sharper parabolas, typically taller individuals (Zorrilla‐Revilla et al. 2024), need to adjust their PLS as close as possible to their OLS due to their limited energy flexibility when walking. This rationale aligns with the fact that older individuals are also typically larger among non‐adults (Bayley 1943; de Onis et al. 2007; Hegde et al. 2020; Khadilkar et al. 2022), leading to higher gross EE, so they should choose energy‐saving strategies. Therefore, the ability of young individuals to maintain speeds different from their OLS without significant energy penalties could have facilitated the dispersal of human groups that included individuals of different ages. This could have contributed to our species' successful colonization of diverse habitats and provided valuable insights into locomotion in children and adolescents from a human ecology and evolutionary perspective.

Although the greater economy of smaller individuals is well established, assessing efficiency requires comparisons and, for this purpose, some studies scale by body mass (Wall‐Scheffler 2022). While standardization by body size is sometimes common, simple ratio standardization (CoT per kg) assumes isometry (exponent = 1); yet most physiological variables do not scale 1:1, so this procedure is not mathematically appropriate, Even so, we will cautiously discuss possible mass‐specific CoT (per kg) consequences, treating them as hypothetical rather than inferential. It is well known that, per kilogram of body mass, smaller individuals may expend more energy than larger ones due to the metabolic costs of growth and maintenance (Hsu et al. 2003; Pontzer et al. 2021; Westerterp 2017). In our context, assuming a given CoT per kg could attenuate the apparent flexibility (because the curvature parameter is rescaled by 1/body mass), and would reduce apparent economy in the smaller participants, while leaving the OLS unchanged. However, total units of metabolism, such as gross CoT (kcal/miles), are the ecologically and evolutionarily relevant units, useful for energy budgets, overall human ecological impact, and the absolute costs of activities (McNab 1999), thus suitable for discussing energy‐saving mechanisms during locomotion in human groups.

### 4.2 The Role of Anthropometric Variables in Locomotion Differences

Conversely, our study presents another novel finding on the potential anthropometric causes behind the differences between PLS and OLS in children and adolescent samples. Specifically, we identified unique contributions from specific anthropometric measurements, such as FL and BIL, to the observed variability in PLS and OLS.

We observed that both FL and BIL contribute to variability in walking speeds in children and adolescents. Previous studies have shown that longer leg length (Alexander 1980; Steudel‐Numbers and Tilkens 2004), and especially longer tibial length (Wall‐Scheffler 2012b), are associated with higher OLS in adults (but see Leurs et al. 2011). However, our study is the first to show a similar trend with FL in populations under 18 years of age. In particular, Mateos et al. (2022) conducted a similar study and found a negative correlation between body weight and OLS, but not between body segments and OLS, in a sample of Western children and adolescents 7–14 years of age. The lack of correlation between body segment dimensions and OLS might be attributed to peak body growth not yet being attained. Perhaps the discrepancy between our findings and those of Mateos et al. (2022) lies in the inclusion of older adolescents in our sample, 18 vs. 14 years of age, respectively. In addition, FL may confer biomechanical advantages that enhance OLS independently of variations in body stature, particularly given that body size was not a significant independent predictor. Other researchers have shown that longer legs (Kramer and Sarton‐Miller 2008; Steudel‐Numbers and Tilkens 2004) and particularly longer FL (Vidal‐Cordasco et al. 2017; Zorrilla‐Revilla et al. 2020) are associated with reduced EE during walking in both adults and non‐adults. However, recent evidence shows an important trade‐off, suggesting that longer FL may also limit energy flexibility during walking, particularly in non‐adult individuals (Zorrilla‐Revilla et al. 2024).

Additionally, we observed a significant correlation between BIL and PLS. Gruss et al. (2017) discussed that broader hips may be associated with a higher PLS. While this result could initially be attributed to factors such as greater body size or older age, neither of these was significant independent predictors of PLS in our analyses. Our findings imply that only BIL may have another biomechanical implication for preferred speed. A possible explanation is that individuals select a pace that prioritizes safety and stability (Majed et al. 2024), which is especially important in certain physiological states, such as in pregnant women (Wu et al. 2004). In this regard, Gast et al. (2019) demonstrated that depending on the walking terrain, safety and stability are prioritized rather than energy efficiency. Wider hips are associated with greater stride length relative to leg length (Gruss et al. 2017) and stability during the gait cycle (Alexander 1991; Elders et al. 1997; Wall‐Scheffler and Myers 2017). In turn, the vertical displacement of the center of gravity is reduced (Wall‐Scheffler 2022; Wall‐Scheffler and Myers 2017). Thus, participants in our study who had broader BIL may have chosen a faster PLS due to increased stability. Conversely, those with narrower hips would choose lower speeds that ensured their walking stability.

### 4.3 Human Ecology Interpretations

Group locomotion promotes cohesion and offers advantages over individual travel; nevertheless, adopting a common speed entails an energetic penalty for those who are farthest from their OLS or who exhibit lower flexibility. There is extensive evidence for this and its implications for the ecology of *Homo sapiens* in Wall‐Scheffler's work, and several studies have shown that adult males typically slow their pace to accommodate slower group members (Bouterse and Wall‐Scheffler 2018; Wagnild and Wall‐Scheffler 2013). Based on our results, we can, from an ecological perspective, consider options for increasing OLS or reducing it in order to walk comfortably (PLS) among children and adolescents within a mixed age and sex group. On the one hand, if the group chooses to walk at the OLS of adult males (which coincides with their PLS), non‐adult individuals would adjust to that group speed with minimal energetic penalty. Adult females, with shorter OLS, would need to increase their pace, although their EE would not rise substantially because they also show some flexibility (Wall‐Scheffler 2012). In energy‐limited contexts, this could be an energetically economical group option that reduces penalties for adult males, who are the least flexible. Conversely, if the group walks at the PLS of non‐adults, which is lower than their own OLS and therefore lower than the OLS of adult males, the latter would be the most penalized. Again, adult females, who exhibit lower OLS than non‐adults (Mateos et al. 2022), would likely be closer to the non‐adults' PLS and thus, incur little penalty relative to walking at their own OLS. It may occasionally be necessary for everyone to increase speed simultaneously because of danger or other requirements; even so, the flexibility and walking speed of non‐adults would mean that this would scarcely affect their daily energy budgets.

Thus, group composition matters: small per‐distance energetic differences at the individual level can accumulate into meaningful group costs over routine travel. Because each person's CoT–speed relation is U‐shaped, deviating from OLS imposes a penalty that rises sharply with the magnitude of the deviation; this penalty is greater for individuals with higher *χ* ^2^ CoT (lower flexibility). Accordingly, it is critical to select a group speed that minimizes the aggregate energetic penalty across all members. Therefore, parties with more flexible walkers tolerate a broader range of group speeds at lower cumulative cost, whereas less flexible parties pay more when pace lies far from members' optima. Across repeated daily trips, even modest per‐mile savings can accumulate into meaningful group‐level differences, allowing greater distance on the same energy budget, reducing foraging time and resource needs, or freeing energy to reinvest in complementary tasks (food processing, resting, childcare, learning), which may indirectly enhance overall reproductive success and survival (group fitness).

Nonetheless, caution is warranted because other physiological and behavioral variables beyond locomotor economy or walking speed also influence gait. Although children may show similar speeds and greater flexibility, they tire more quickly (lower stamina), thermoregulate less effectively throughout a walk, or are frequently distracted while displacing, since at times learning rather than displacement is the goal (Bombjaková et al. 2020; Carrier 1996; Lew‐Levy and Boyette 2024; McClain and Vandermaas‐Peeler 2016; Zorrilla‐Revilla 2022; Zorrilla‐Revilla et al. 2024).

### 4.4 Evolutionary Perspective

Based on the findings presented in this study, it can be inferred that as *Homo sapiens* grow and increase their stature‐related body dimensions, their walking speeds tend to rise as well. This applies to both the freely chosen speed (PLS) and the most efficient based on EE per distance traveled (OLS). Nevertheless, these enhancements come with a trade‐off: related to bigger body size, older individuals in our sample experienced a reduction in energy flexibility when they deviated from their OLS and opted for a preferred speed that may not have aligned with their most efficient one. This trade‐off can be key because, after human gait and economy of locomotion mature at around 7 and 8 years of age (Froehle, Nahhas, et al. 2013; Kramer 1998), young girls and boys improve their motor and complex skills through movement (Berghänel et al. 2015). These skills are critical for future survival, particularly in subsistence economies such as hunter–gatherer societies. Therefore, energy‐saving mechanisms that enable learning by doing at low cost could not only improve their fitness, but also the group that supports them, especially mothers (Zorrilla‐Revilla et al. 2021; Zorrilla‐Revilla 2022).

These factors may have held significant importance in past hominin groups constrained by keeping a narrow balance between energy intake and EE. For example, based on the findings of Zorrilla‐Revilla et al. (2024), we can hypothesize that previous “wide Homo” body bauplan species (Arsuaga et al. 2015; Carretero et al. 2012; García‐González et al. 2024), characterized by an earlier and faster body growth trajectory (García‐González et al. 2009), would have faced greater energetic disadvantages while walking compared to *Homo sapiens* of the same age. Hence, if their PLS was higher due to wider BIL derived from a mediolateral (ML) wide biotype (Arsuaga et al. 2015; García‐González et al. 2024), and their OLS remained relatively similar due to their FL compared to a non‐adult *Homo sapiens* , they could not have chosen comfortable speeds very far from their optimum, reducing the chances of expending energy on other physiological allocation targets (growth and maturation, maintenance, physical activity). Although, wider hips would indeed mitigate the high cost of moving a larger body while walking (Vidal‐Cordasco et al. 2017) by reducing the number of steps at a given speed or the activity of the adductors (Gruss et al. 2017; Wall‐Scheffler et al. 2010). However, this restricted repertoire of suboptimal speeds at which other non‐adult individuals of species other than *Homo sapiens* could walk would have imposed a greater disadvantage when moving within groups composed of individuals of different ages and sexes, as evidenced by studies of fossil footprints (Altamura et al. 2023; Ashton et al. 2014; Mayoral et al. 2021). An example of this would be an increase in the total EE of the group, which would need to be compensated for by either increasing foraging rates or decreasing foraging ranges and/or mobility distances. An alternative strategy to mitigate this energy burden might involve forming groups consisting mainly of non‐adults (Duveau 2021; Duveau et al. 2019). However, such a composition could also decrease survival rates against predators (Duveau 2021). These aspects could have played a crucial role in species competition and survival dynamics in the past.

### 4.5 Limitations and Considerations

There are some limitations of our experimental study that we should acknowledge. All volunteers came from a Western industrialized and urban society and not from human groups whose way of life is based on hunting and gathering. Additionally, all participants walked on a treadmill instead of a natural surface. While treadmill use can influence the sense of stability (Malatesta et al. 2017), this limitation was consistent across all participants, minimizing its impact on comparative results. Future research should incorporate samples from non‐industrialized societies and real‐world walking conditions, such as Venkataraman et al. (2018) or Holowka et al. (2022), to validate these findings under more ecologically relevant conditions. Hip width was represented by the BIL measurement in this study; however, other hip measurements not assessed here could affect outcomes differently. Beyond these limitations and considerations, our study offers several notable strengths. We explored a rarely studied population in locomotor energetics, focusing on children and adolescents at different stages of growth and development. Detailed anthropometric data allowed for the identification of key morphological variables influencing both optimal and preferred walking speeds. By situating these results within an evolutionary and ecological context, our study adds depth to understanding energy use and mobility in non‐adult humans. This is the first study to address both variables, and their divergence, from an evolutionary perspective, proposing hypotheses about how these dynamics may have operated in other hominin species.

## 5 Conclusion

Based on our results, we can suggest that PLS and OLS are not equivalent, at least in the age groups examined. It seems that minimizing the EE per unit of distance (COT) did not fully represent their goal when selecting a comfortable walking pace, given the wide repertoire of suboptimal speeds and certain anthropometric factors that allowed them to deviate from their optimal expenditure. However, when body size increases, older adolescents tend to choose preferred speeds closer to the optimal ones, ensuring locomotion efficiency. While this may confer a significant advantage, it could pose a challenge for species that have different growth and development patterns than *Homo sapiens* , particularly in the context of species competition.

## Author Contributions

**Guillermo Zorrilla‐Revilla:** conceptualization, formal analysis, investigation, writing – original draft, writing – review and editing, methodology. **Olalla Prado‐Nóvoa:** investigation, methodology, writing – review and editing. **Kevin P. Davy:** investigation, methodology, resources, writing – review and editing. **Rebeca García‐González:** supervision, writing – review and editing, funding acquisition. **Eleni Laskaridou:** methodology, writing – review and editing. **Kristen R. Howard:** methodology, writing – review and editing. **Elaina L. Marinik:** methodology, writing – review and editing. **José Miguel Carretero:** writing – review and editing. **Stella L. Volpe:** funding acquisition, methodology, project administration, supervision, resources, writing – review and editing.

## Ethics Statement

The experimental study was approved by the Institutional Review Board at Virginia Polytechnic Institute and State University (Virginia Tech) (IRB #23–324).

## Consent

Written informed consents were obtained from all legal guardians and assent was obtained from children and adolescents. No photographs or other supporting material were obtained.

## Conflicts of Interest

The authors declare no conflicts of interest.

## Data Availability

The data that support the findings of this study are available on request from the corresponding author. The data are not publicly available due to privacy or ethical restrictions.
